# Structural and functional characterization of sulfurtransferase from *Frondihabitans* sp. PAMC28461

**DOI:** 10.1371/journal.pone.0298999

**Published:** 2024-03-25

**Authors:** Hackwon Do, Dieu Linh Nguyen, Yong-Yoon Ahn, Yewon Nam, YoonJi Kang, HoeJung Oh, Jisub Hwang, Se Jong Han, Kitae Kim, Jun Hyuck Lee

**Affiliations:** 1 Research Unit of Cryogenic Novel Material, Korea Polar Research Institute, Incheon, Korea; 2 Department of Polar Sciences, University of Science and Technology, Incheon, Korea; 3 Division of Life Sciences, Korea Polar Research Institute, Incheon, Korea; Chung-Ang University, REPUBLIC OF KOREA

## Abstract

Sulfurtransferases transfer of sulfur atoms from thiols to acceptors like cyanide. They are categorized as thiosulfate sulfurtransferases (TSTs) and 3-mercaptopyruvate sulfurtransferases (MSTs). TSTs transfer sulfur from thiosulfate to cyanide, producing thiocyanate. MSTs transfer sulfur from 3-mercaptopyruvate to cyanide, yielding pyruvate and thiocyanate. The present study aimed to isolate and characterize the sulfurtransferase *Fr*ST from *Frondihabitans* sp. PAMC28461 using biochemical and structural analyses. *Fr*ST exists as a dimer and can be classified as a TST rather than an MST according to sequence-based clustering and enzyme activity. Furthermore, the discovery of activity over a wide temperature range and the broad substrate specificity exhibited by *Fr*ST suggest promising prospects for its utilization in industrial applications, such as the detoxification of cyanide.

## Introduction

Sulfurtransferases, also known as rhodaneses, facilitate the transfer of sulfur from a donor molecule to a thiophilic acceptor. The rhodaneses have been studied primarily for their role in detoxifying cyanide, a significant environmental pollutant and toxic chemical present in various industrial and natural environments. In mice, rhodaneses catalyze the transfer of sulfur from sodium thiosulfate to cyanide, resulting in the conversion of cyanide into thiocyanate, a comparatively less toxic compound [1–3].

Sulfur donors, such as aliphatic compounds like ethanethiosufonate, oxidized glutathione (GSSH), and 3-mercaptopyruvate, along with thiophilic acceptors like sodium sulfite, cysteine, and free glutathione (GSH), have been identified as substrates for rhodaneses [4, 5]. These enzymes are involved in various biological processes, including sulfur and selenium metabolism, as well as in the synthesis and repair of iron-sulfur proteins in plants, animals, and microbes. Additionally, owing to their ability to detoxify cyanide, rhodaneses have shown potential for medical and industrial applications. For example, the human pathogen *Pseudomonas aeruginosa* produces hydrogen cyanide (HCN) as a virulence factor via HCN synthase activity [6]. Transgenic flies that overexpress bovine rhodaneses have been found to be resistant to *P*. *aeruginosa* strains that produce cyanide [7]. Another example involves the detoxification of cyanide by engineered *Escherichia coli*. Cipollone et al. demonstrated that a genetic system that overexpresses rhodaneses at high levels could effectively reduce toxic cyanide levels, making it suitable for cyanide transformation in natural environments [3].

Rhodaneses (EC 2.8.1.) can be categorized into two subgroups based on the type of sulfur donor utilized: (1) thiosulfate sulfurtransferases (TSTs, EC 2.8.1.1) and (2) 3-mercaptopyruvate sulfurtransferases (MSTs, EC 2.8.1.2). Structural studies have indicated that both TSTs and MSTs share a similar catalytic mechanism involving two distinct intermediates, in which the enzymes cycle between a sulfur-free state and a persulfurated intermediate on a catalytic cysteine residue. To shed light on this aspect, a study was conducted on *Eco*SseA from *E*. *coli*, which is classified as an MST. A notable difference between the two subgroups is the amino acid composition of the active site surrounding the catalytic cysteine. MSTs contain the CGS(T)GVTA sequence, while TSTs possess the CRKGVTA sequence. Mutations (e.g., Ser to Ala or Lys) at the third amino acid of the catalytic cysteine in *Eco*SseA alter substrate preference, highlighting the significance of this loop in substrate binding and recognition [8]. However, the detailed mechanisms governing substrate preference and binding have yet to be elucidated.

In this study, we aimed to determine the crystal structure of *Fr*ST isolated from *Frondihabitans* sp. PAMC28461. *Fr*ST was found to be catalytically active toward thiosulfate instead of 3-mercaptopyruvate, as revealed by structural and biochemical analyses. Structural analysis and comparison with known homologues identified distinct features that differentiated the two subgroups of rhodaneses.

## Results and discussion

### Sequence analysis and comparison of *Fr*ST with TST and MST groups

**Fig 1:**
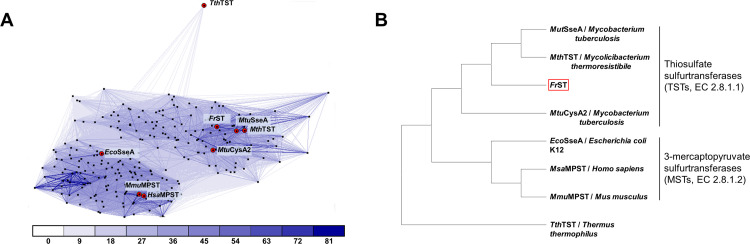
Sequence and structure comparison of *Fr*ST with TST and MST groups. (A) Sequences of orthologues from different species were used for CLANS clustering [9]. Sulfurtransferases on panel A are represented by red dots, and the connecting lines between them indicate the level of sequence similarity. Darker and shorter lines indicate higher sequence similarity. The connections with P-values better than 1e-x (where x is a number below) are depicted in the corresponding color. (B) The phylogenetic tree of characterized sulfurtransferases, including *Fr*ST, was generated using MEGA X software with 1000 bootstrap replicates. The tree was constructed based on amino acid sequences to assess the evolutionary relationships between different sulfurtransferases.

TSTs (EC 2.8.1.1) and MSTs (EC 2.8.1.2) are subgroups of sulfurtransferases that depend on sulfur donors. Although amino acids are well conserved throughout sulfurtransferases from various species, including catalytic cysteine at the active site, the two subgroups were distinguishable through phylogenetic tree and clustering analysis (Fig 1A). Clustering analysis using CLANS [9] showed that *Fr*ST belongs to the TST group, which includes other thiosulfate sulfurtransferases, such as *Mtu*SseA from *Mycobacterium tuberculosis*, *Mth*TST from *Mycobacterium thermoresistible*, and *Mtu*CysA2 from *M*. *tuberculosis*. Additionally, phylogenetic tree analysis of these sulfurtransferases further confirmed the classification of *Fr*ST within the TST subgroup. Among the sulfurtransferases analyzed, *Mut*SseA was most closely related to *Fr*ST in terms of sequence similarity (Fig 1B). These findings suggest that *Fr*ST is a TST subgroup member and likely exhibits a preference for thiosulfate as a substrate.

### Enzymatic activity of *Fr*ST

To investigate the substrate preference of *Fr*ST, enzyme activity was measured using various substrates and cyanide as the sulfur acceptor (Table 1 and S1 Fig). The results revealed that *Fr*ST had an activity toward both thiosulfate and 3-mercaptopyruvate. Moreover, *Fr*ST exhibited a preference for broad substrates that could be categorized into two groups: a sulfoxide-containing group and a carboxyl-containing group with three carbons. The sulfoxide-containing groups, including sodium benzenethiosulfonate, sodium methanethiosulfonate, and thiosulfate, displayed a relatively high affinity for the enzyme compared to the carboxyl-containing group. The catalytic efficiency (*k*_*cat*_/*K*_*m*_) for sulfur transfer from sodium benzenethiosulfonate or sodium methanethiosulfonate to cyanide was 66 or 12.8 times higher, respectively, than that from thiosulfate to cyanide. The presence of a benzene ring in sodium benzenesulfonothioate and a methyl group in sodium methanesulfonothioate suggests that hydrophobic interactions between the substrate and *Fr*ST play an important role in catalytic activity.

**Table 1:** Kinetic parameters determined for *Fr*ST with various sulfur donors.

Donor	Kinetic parameters^*^			
	*K*_*m*_ (*mM*)	*V*_*max*_ (*μmol min*^*–1*^ *mg*^*–1*^)	*k*_*cat*_ (S^–1^)	*k*_*cat*_ /*K*_*m*_ (M^–1^ S^–1^)
Sulfoxide-containing substrates				
Sodium benzenethiosulfonate	6.1 ± 0.9	7807 ± 581	445	73.0 × 10^3^
Sodium methanethiosulfonate	6.0 ± 0.3	1493 ± 111	85	14.2 × 10^3^
Sodium thiosulfate	6.3 ± 0.2	128.2 ±2.8	7.1	1.1 × 10^3^
Carboxyl-containing substrates				
Sodium 3-mercaptopyruvate	13.9 ± 2.6	149.5 ±12.2	8.6	0.61 × 10^3^
3-mercaptopropionic acid	10.7 ± 1.3	43.7 ± 8.9	2.6	0.25 × 10^3^
3-mercaptoisobutyric acid	4.1 ± 0.4	17.8 ± 0.4	0.99	0.24 × 10^3^
4-mercaptobutyric acid	0 ± 0	0 ± 0	0 ± 0	0 ± 0
Thioglycolic acid	0 ± 0	0 ± 0	0 ± 0	0 ± 0

^*^mean ± standard deviation based on triplicate experiments for all substrates.

In the case of carboxyl-containing substrates, *Fr*ST exhibited transferase activity against three of the five substrates, including sodium 3-mercapto pyruvate3-mercaptopropionic acid, and 3-mercaptoisobutyric acid (Table 1). Among these three substrates, sodium 3-mercaptopyruvate exhibited slightly higher catalytic activity compared to 3-mercaptopropionic acid and 3-mercaptoisobutyric acid.

It was previously suggested that sodium 3-mercaptopyruvate is the sole sulfur donor substrate for MST [10]. To further confirm *Fr*ST’s activity, we conducted mass spectrometry analysis with 3-mercaptopropionic acid and 3-mercaptoisobutyric acid. The resulting spectra displayed well-resolved peaks for propionate and isobutyrate with great peak shapes (S2 Fig). The retention times for propionate and isobutyrate were measured at 1.14 minutes and 1.17 minutes, respectively, as reaction products of 3-mercaptopropionic acid and 3-mercaptoisobutyric acid catalyzed by *Fr*ST. In the mass spectrometry data, signals corresponding to the deprotonated molecular ions of propionate [*m/z* 73.0293; C_3_H_5_O_2_] and isobutyrate [*m/z* 87.0449; C_4_H_7_O_2_] were detected, respectively. Furthermore, we identified the major peaks for the substrates, indicating that the conversion rate of *Fr*ST against these substrates is not efficient. These findings align with the results obtained from the colorimetric assay. However, no enzyme activity was observed when using 4-mercaptobutyric acid and thioglycolic acid as substrates. Notably, 4-mercaptobutyric acid and thioglycolic acid had four and two carbons in their backbones, respectively, whereas all other carboxyl-containing substrates were composed of three carbons. This indicates that *Fr*ST prefers carboxyl-containing substrates of specific sizes. In conclusion, the sequence analysis and enzyme activity of *Fr*ST showed that *Fr*ST has high sequence similarity with the TST group and high activity against thiosulfonate and thiosulfate substrates.

**Fig 2:**
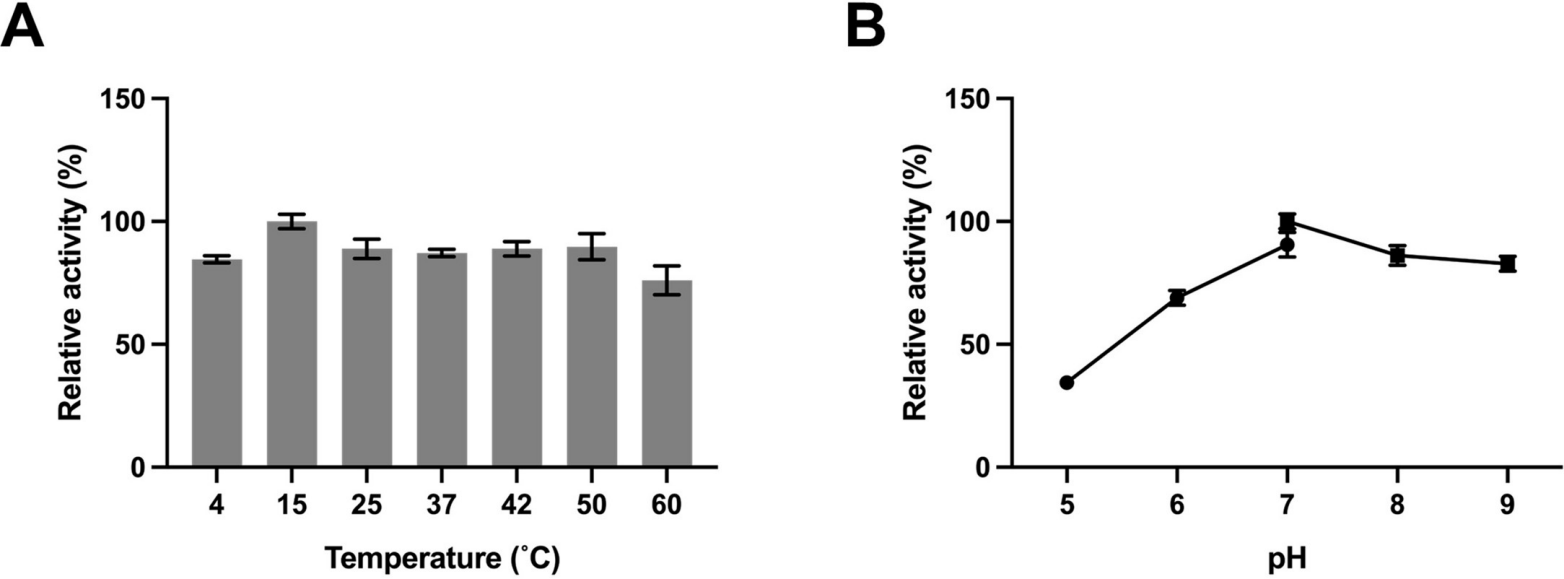
**Characterization of *Fr*ST.** (A) The temperature effects on enzyme activity were determined using sodium thiosulfate as sulfur donor in Tris-HCl buffer (pH 8.0). (B) The pH effects on enzyme activity were determined in different buffers: 50 mM citrate phosphate buffer (pH 5.0–7.0) and 100 mM Tris-HCl buffer (pH 7.0–9.0). All of the assays were performed at 25°C using sodium thiosulfate as a sulfur donor. The highest activity was determined as 100%. Data are presented as the mean of three individual experiments.

The influence of temperature and pH on *Fr*ST activity was investigated using thiosulfate as the sulfur donor, as depicted in Fig 2. *Fr*ST exhibited the highest activity at 15°C. Notably, *Fr*ST demonstrated a broad range of thermal activities, as its enzyme activity remained above 85% at various temperatures. In particular, *Fr*ST retained activity even at a low temperature of 4°C, with activity exceeding 80% of its maximum level. To examine the effect of pH on enzyme activity, the reaction cocktail was incubated at different pH values using 50 mM citrate phosphate for pH 5.0–7.0 and 100 mM Tris-HCl for pH 7.0–9.0, and the resulting product was measured. The data indicated that *Fr*ST displayed maximum activity at pH 7.0.

### Overall structure of *Fr*ST

To understand the structural features of *Fr*ST, its three-dimensional structure was determined by X-ray crystallography. The crystal structures were solved by the molecular replacement method using a sequence-based model obtained from SWISS-MODEL [11]. Within the crystal, each asymmetric unit contained two subunits, designated A and B. After refinement, the final protein model of *Fr*ST exhibited a crystallographic R-factor of 23% and a free R-factor of 26%. The model comprised 302 residues and 339 water molecules (S1 Table). One or two amino acids in the N-terminal and C-terminal regions of protein were in contact with the solvent and were not discernible in the electron density map. However, the remaining residues were successfully traced based on the electron density, except for a loop of Thr188–Glu194 (later termed as 8α-η2 loop region) in subunit B, indicating the high flexibility of this loop region.

**Fig 3:**
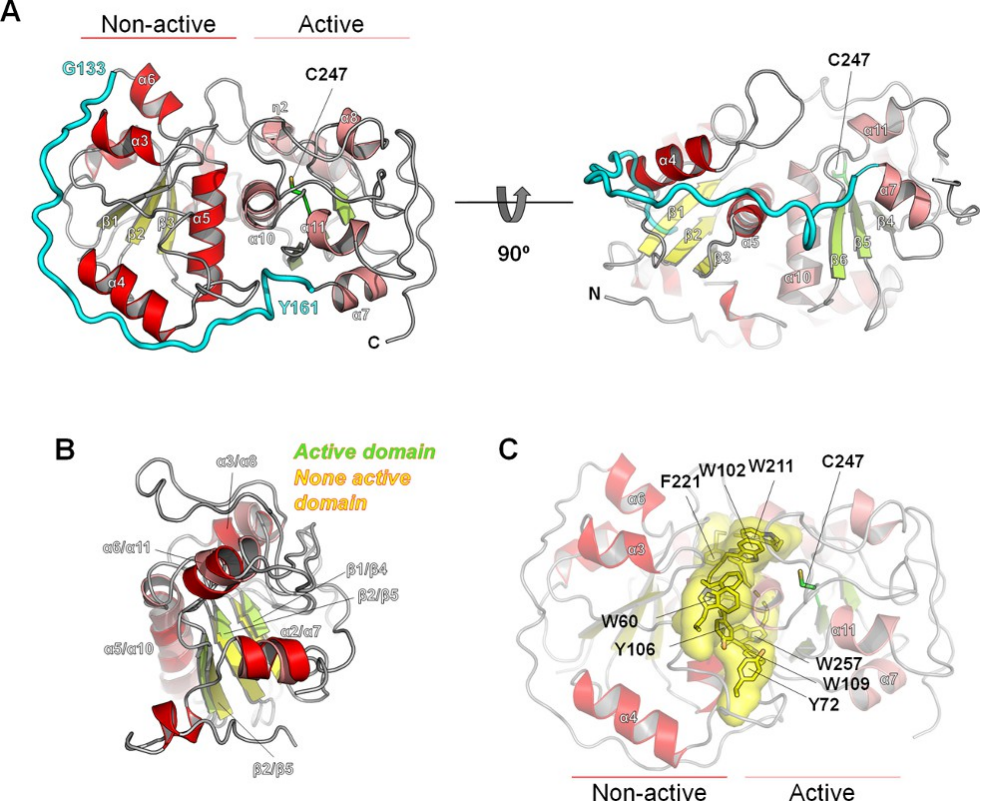
The overall structure of *Fr*ST. (A) The overall structure of *Fr*ST is depicted in a ribbon diagram, providing a front view on the left and a 90° rotated view on the right. The non-active domain is represented by red helices and yellow strands, while the active domain is shown in pink helices and green strands. The catalytic cysteine at the active site is visualized as a green stick. The long loop that connects the non-active N-terminal domain and the active C-terminal domain is displayed in cyan. (B) A superimposition of the N-terminal non-active domain and the C-terminal active domain is shown, highlighting the similarity between the two domains and illustrating their relative arrangement within the *Fr*ST protein. (C) The aromatic residues situated between the two domains are indicated by yellow sticks and surfaces.

Each subunit of *Fr*ST comprised 11 α-helices, two 3_10_ helices, and six β-strands and was divided into two distinct domains: the N-terminal rhodanese fold domain (non-active domain) and the C-terminal rhodanese fold domain (active domain) (Fig 3A). The α-helices surrounded three β-strands and formed a central parallel β-sheet within each domain. These two domains were connected by a long loop (from Gly133 to Tyr161) that wrapped around the inactive N-terminal domain (Fig 3A). Despite being structurally identical, with a root-mean-square deviation (RMSD) value of 3.7, only the C-terminal domain contained the catalytic residue Cys247, located at the end of the central β5 (Fig 3B). The two domains interacted strongly with each other through specific inter-residue interactions. The aromatic ring residues in the interdomain region formed a connected circle, contributing to hydrophobic interactions via a combination of π-stacking and semi-π interactions (Fig 3C). These interactions appear to play a crucial role in the formation of the two domains rather than in the long loop highlighted in cyan in Fig 3A.

### *Fr*ST forms a dimer

**Fig 4:**
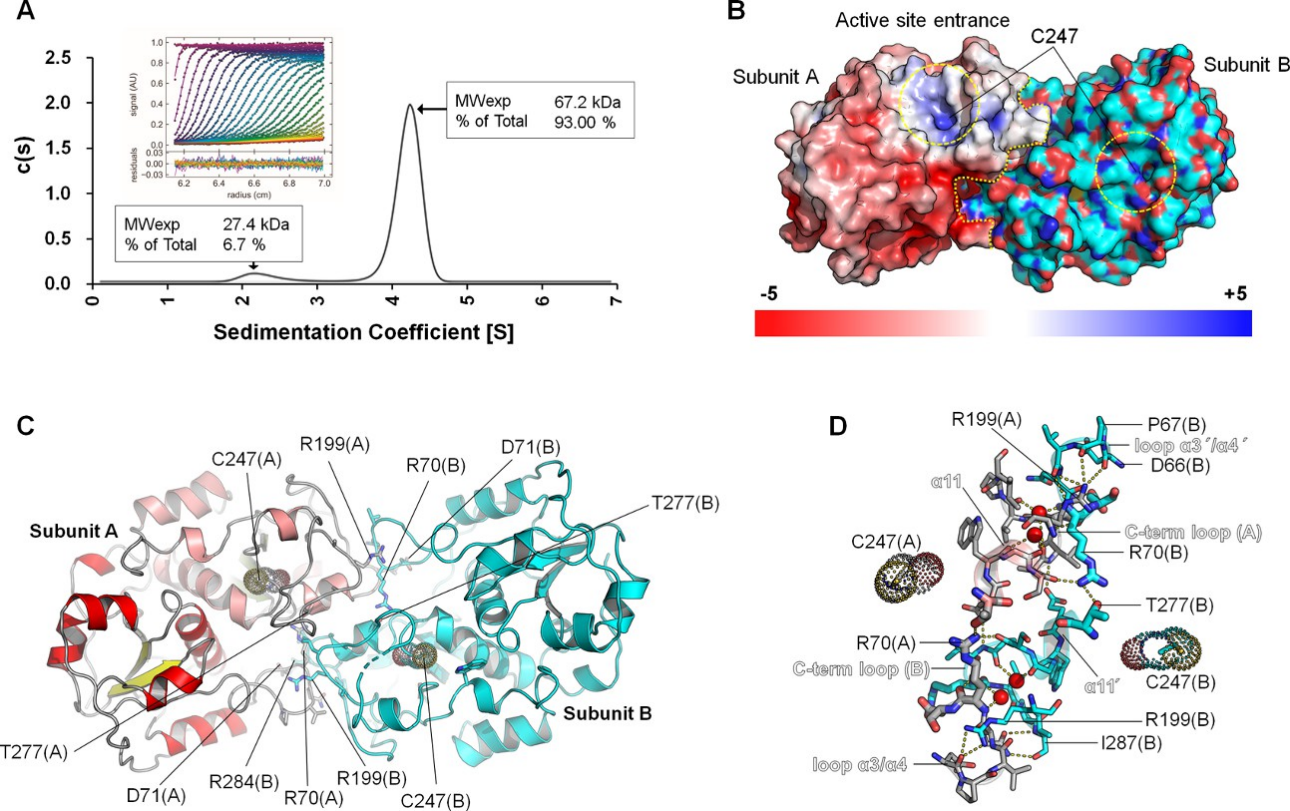
Dimerization of *Fr*ST. (A) The dimeric form of *Fr*ST was analyzed by sedimentation velocity analytical ultracentrifugation. Sedimentation profiles were scanned every 6 min at 280 nm and colored with progressive rainbow colors using the GUSSI program (inset figure). The experiment was performed in 20 mM Tris-HCl (pH 8.0) and 200 mM NaCl at 20°C using ProteomeLab XL-A (Beckman Coulter, Inc., Brea, CA, USA). (B) The surface representation of *Fr*ST (subunit A) depicted with an electrostatic surface is shown. The boundaries of the protein and the active sites are indicated by yellow dotted lines, providing insight into the distribution of electrostatic charges on the protein surface. (C, D) The dimer interface of *Fr*ST is shown, highlighting the residues involved in the interaction between subunits (stick residues).

*Fr*ST exists as a dimer composed of subunits A and B in solution, as confirmed by analytical ultracentrifugation (AUC). This dimeric arrangement was consistent with the assembly observed in the crystallographic analysis. AUC data revealed that the calculated molecular weight of *Fr*ST was approximately 67.2 kDa, which closely matched the theoretical mass of the *Fr*ST dimer (66.6 kDa) (Fig 4A). The dimer interface was formed by the interaction between the C-terminal helix α11 of one subunit and α11’ of the second subunit (Fig 4B). This interface was primarily stabilized by hydrogen bonds. Furthermore, the loop region between α3 and α4, as well as the C-terminal loop of both subunits, contributed to the stability of the dimer interface. Notably, two arginine residues, Arg70 and Arg199, located in the loops of each subunit, played a significant role in dimerization by interacting with their counterparts in the neighboring subunit (Fig 4C and 4D).

### Active site of *Fr*ST

**Fig 5:**
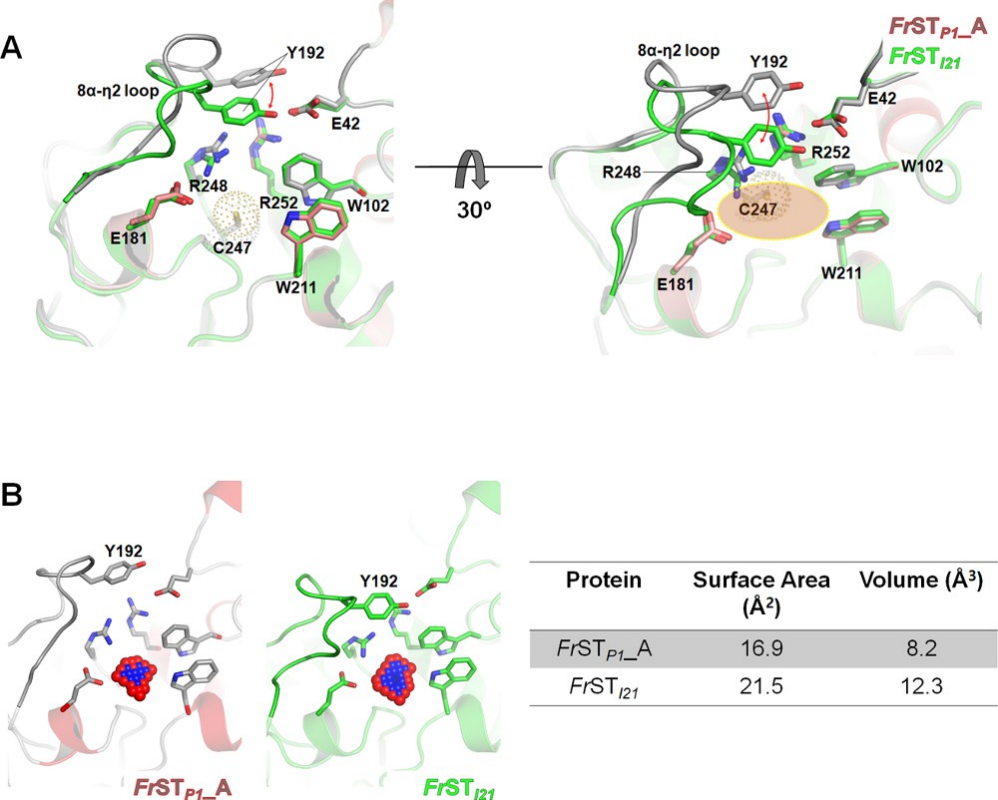
Different conformations of the 8α-η2 loop region are observed within *Fr*ST. (A) Two subunits from the crystallographic asymmetric unit are superimposed to represent the structural change, shown at different angles of view. The active site is marked with an orange oval. (B) The surface area and volume of the active sites in each subunit were calculated using the ParKVFinder with 1.4 Å of a default probe radius.

The active site of *Fr*ST was composed of Trp102, Val176, Arg177, Glu181, Trp211, Arg248, and Arg252, which surrounded catalytic Cys247. In particular, the cavity lined with Arg177, Trp211, Arg248, and Arg252 provided a positively charged surface, which may facilitate a favorable environment for accommodating substrates (Fig 5A).

During the crystal structure determination, we also solved another crystal structure of *Fr*ST, which belongs to the *I2*_*1*_ space group and is annotated as *Fr*ST_*I21*_. The overall structure of both structures is almost identical, evidenced by an RMSD of 0.284 Å (Cα). However, when *Fr*ST_*I21*_ could closely resemble *Fr*ST_*P1*_, a distinct conformational difference was observed in the 8α-η2 loop region at the top of the active site. In subunit A of *Fr*ST_*P1*_, the 8α-η2 loop region (residues Ala186–Ala198) adopted an open conformation, as supported by clear electron density in the corresponding map (S3 Fig). Glu195 formed hydrogen bonds with Arg248 and Arg252 from the active site loop, while Tyr192 interacted with neighboring Arg252 through a cation-π interaction (Fig 5A). In contrast, in *Fr*ST_*I21*_, Tyr192 tilted toward the active site and engaged in cation π-interaction with Arg252 as well as a hydrogen bond with Glu42. This arrangement resulted in the formation of a cavity for substrate binding, unlike that in *Fr*ST_*P1*_ (Fig 5A). The swinging motion of Tyr192 induced changes in the active site cavity volume of *Fr*ST. Volume analysis using ParKVFinder 3.0 [12] revealed that the volume of the active site in *Fr*ST_*P1*_was 8.2 Å^3^, whereas it was 12.3 Å^3^ in *Fr*ST_*I21*_ (Fig 5B). Similar conformational variations in the 8α-η2 loop region were also observed in other rhodaneses, such as *Mtu*SseA and *Mth*TST (S4 Fig) [13]. The diverse amino acid composition and structural flexibility, as evidenced by weak electron density in the 8α-η2 loop region, suggests that this region is likely involved in an open and closed mechanism that facilitates substrate recognition and selectivity.

**Fig 6:**
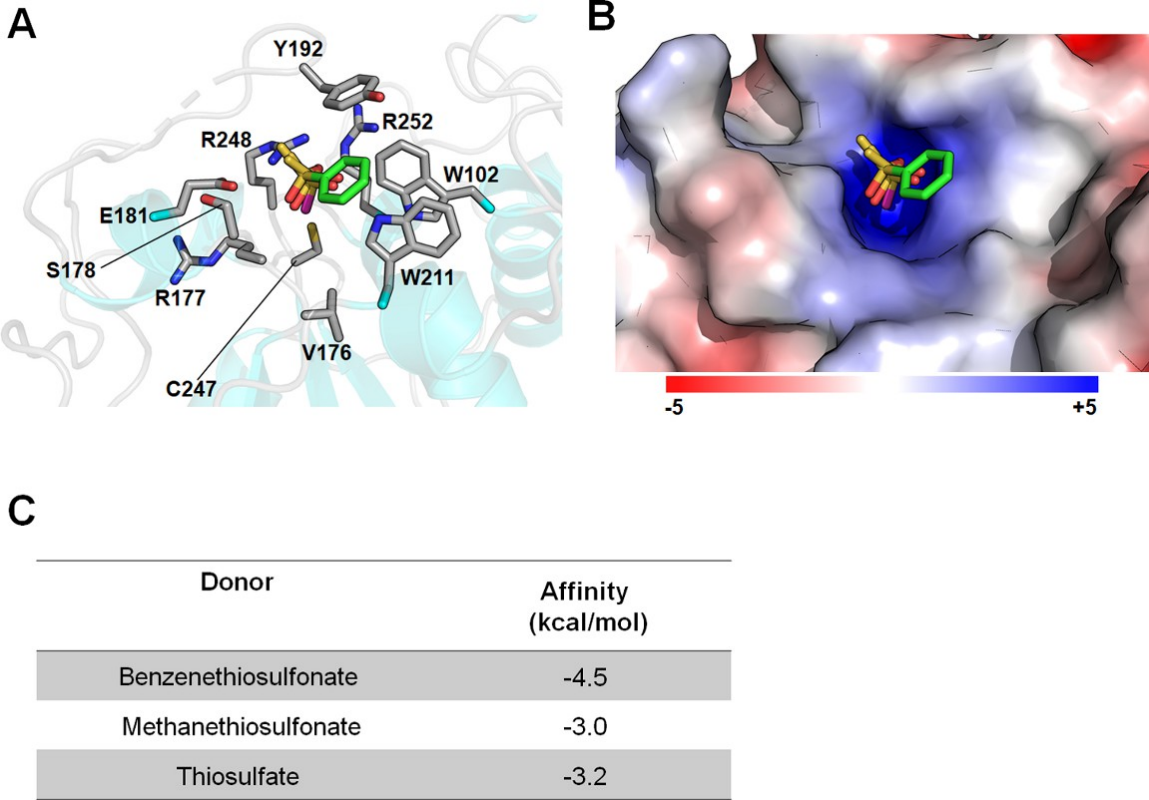
The active site arrangement and surface charge distribution of *Fr*ST. (A) Docking simulation using benzenethiosulfonate (green), methanethiosulfonate (pink), and thiosulfate models with *Fr*ST are depicted. Side chains of active site residues of *Fr*ST are shown in the sticks. (B) The electrostatic surface potential of *Fr*ST with the same orientation as in panel A. The surface potential is shown from -5 KT/e in red to +5 KT/e in blue. (C) The binding affinities of the substrates resulting from the docking simulations are shown.

To understand the substrate recognition mechanism of *Fr*ST, a docking experiment was conducted using substrate models used for activity measurements. As shown in Fig 6, three substrates, namely benzenethiosulfonate, methanethiosulfonate, and thiosulfate, were docked at the active site of *Fr*ST_*I21*_. The tetrahedral sulfonate or sulfide of each substrate was positioned at the active site in a similar conformation and interacted with the side chains of Glu181 and Arg248 as well as the backbend of Ser178 via hydrogen bonds (Fig 6A and 6B). As hypothesized from the enzyme activity, benzenethiosulfonate showed a higher binding affinity than the substrates (Fig 6C). The benzene ring of benzenethiosulfonate was located between Tyr192 and Trp211 within a possible range of hydrophobic interactions.

### Comparison of *Fr*ST with the MST subgroup

**Fig 7:**
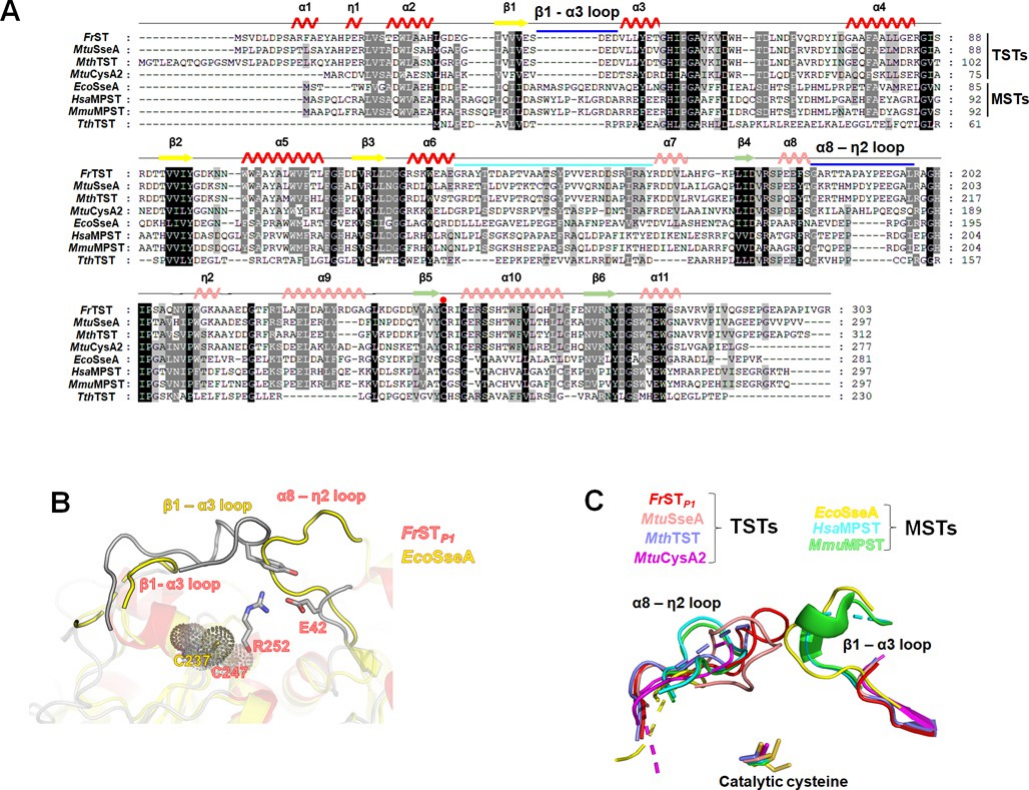
Comparison of loops from TST and MST groups. (A) A multiple sequence alignment was conducted to compare the amino acid sequences of sulfurtransferases. The secondary structure elements of *Fr*ST are depicted above the aligned sequences. In the alignment, additional amino acids inserted in sulfurtransferases from the TST or MST subgroup are marked with blue bars. The connecting loop between the two domains is highlighted in cyan. (B) Superimposition of *Fr*ST with *Eco*SseA from the MST subgroup was performed to compare their structures. The loop segment from residue 26 to 37 in *Eco*SseA and the 8α-η2 loop in *Fr*ST is highlighted in the superimposed structure. (C) The β1–α3 loop and 8α–η2 loop, covering the active site from TST and MST groups, are depicted with different color codes.

As shown in the multiple sequence alignment, sequential features specific to each subgroup were also found (Fig 7A). The TST subgroup lacked amino acids in the loop region between β1 and α3 and had additional amino acids at the 8α-η2 loop of *Fr*ST. The sequence features were exhibited in the structure. Structural comparison of *Fr*ST with *Eco*SseA (Protein Data Bank code: 1URH) as a representative structure for the MST subgroup showed that the two loops were closely located at the cap of the active site and that the additional amino acid from each long loop covered the active site. The β1-α3 loop of *Fr*ST extruded into the center of the substrate-binding site and interacted with Arg252 of the active site loop 10 Å away from Cys247. Similarly, the TST subgroup had an additional five amino acids in the 8α-η2 loop region (8α-η2 loop of *Fr*ST) to replace the role of covering the active site (Fig 7B). However, in *Eco*SseA from the MST subgroup, this region (26–37 segment) was oriented toward the active site to cover it with additional amino acids (Fig 7C). This result further confirmed that *Fr*ST belongs to the TST subgroup. Because each subgroup has a different substrate preference, the loop covering the active site of the two subgroups may participate in substrate recognition at a proximate location [14].

## Conclusions

In the present study, we successfully isolated and characterized the sulfurtransferase *Fr*ST from *Frondihabitans* sp. PAMC28461. Analyses of amino acid sequences and enzymatic activity indicated that *Fr*ST has a substrate preference for sulfoxide-containing groups, indicating its classification as a TST subgroup of sulfurtransferases. Additionally, by conducting docking simulations with thiosulfate and thiosulfonate substrates, we predicted that hydrophobic interactions involving Trp102 and Trp211 would be crucial for sulfur transfer in *Fr*ST from sodium benzenethiosulfonate. *Fr*ST also showed catalytic activity against carboxyl-containing substrates, indicating its broad substrate specificity. Furthermore, *Fr*ST retained its activity over a wide range of temperatures and pH values. When comparing the structure with that of MSTs, we found that each subgroup possessed a distinct loop that covered the active site, suggesting that this loop may be involved in distinguishing substrates for sulfurtransferases. Although further structural investigations of *Fr*ST complexed with active substrates and mutational studies are required, these findings provide structural information and insights into the characteristics of *Fr*ST as a representative of the TST subgroup.

## Materials and methods

### Recombinant protein expression and purification

The gene encoding the sulfurtransferase from *Frondihabitans* sp. PAMC28461 was synthesized and cloned into a pET28a expression vector (Novagen). The pET28a:*FrST* construct was then transformed into BL21 (DE3) strains of *E*. *coli*. Transformed cells were induced to express the protein by adding 1 mM isopropyl β-D-1-thiogalactopyranoside when the culture reached an optical density of 0.5 at 600nm. Protein expression was measured for 20 h at 25°C.

After the expression period, the cells were harvested and lysed by sonication. A sequential two-step purification process was used to purify the proteins. First, the soluble fraction of the cell lysate was loaded onto a column containing a resin with an affinity for the His-tag fused to the recombinant protein. The column was washed with NPI30 buffer, which is a sodium phosphate buffer containing 30 mM imidazole. Subsequently, the His-tag was cleaved from the purified protein using thrombin (0.5 U/mg of protein). This step removes the His-tag, resulting in the generation of an untagged protein of interest. Finally, the protein was further purified using size-exclusion chromatography with a HiLoad 26/600 Superdex 200 pg column. The purity and quantity of the recombinant proteins were analyzed using SDS-PAGE and NanoDrop spectroscopy, respectively.

### Crystallization, data collection, and refinement

Approximately 15 mg/ml recombinant *Fr*ST was crystallized using the sitting-drop vapor diffusion method. The *Fr*ST crystals grew under conditions consisting of 10% w/v PEG 20,000, 20% v/v PEG MME 550, 0.03 M magnesium chloride, calcium chloride as divalent cations, and 0.1 M MOPS/HEPES-Na pH 7.5 at room temperature for one week. A single flat crystal without cryoprotectant was mounted on a goniometer equipped with a nitrogen stream. The data were collected at beamline 5C at Pohang Light Source in Korea, using an oscillation range of 1°, and processed using XDS [15]. Models from the SWISS-MODEL [11] and molecular replacement [16] methods were applied to obtain the *Fr*ST phase. Then, a combination of Coot [17], Autobuild, and phenix.refine from Phenix [18] was used for the iterative model building of the *Fr*ST structure. The quality of the final model was verified using Molprobity [19]. The Ramachandran plot indicated that the model was built with 93.45% of the residues in the favored regions and 2.52% of the residues in the disallowed regions. The coordinates and structure factors of *Fr*ST were deposited in the Protein Data Bank RCSB under accession codes: 8K55 (*Fr*ST_*P1*_) and 8K57 (*Fr*ST_*I21*_). X-ray data collection and refinement statistics are provided in S1 Table. All structure-related figures were generated using PyMOL [20].

### Analytical ultracentrifugation

To investigate the oligomerization state of *Fr*ST, sedimentation velocity analytical ultracentrifugation experiments were performed using a ProteomeLab XL-A (Beckman Coulter, Fullerton, CA, USA) analytical ultracentrifuge equipped with a scanning UV/Vis detection system. 0.4 mL of protein sample was loaded alongside 0.42 mL of a reference buffer (20 mM Tris-HCl, pH = 8.0, 200 mM NaCl). The analysis was conducted at 20°C and 42,000 RPM, with scans taken every 6 minutes until complete sedimentation was achieved. Sedimentation profiles were monitored at 280 nm, and the data were analyzed using SEDFIT software.

### Calculation of surface pockets

The ParKVFinder server was used to calculate the volume of the active sites [12]. The default probe radius of 1.4 Å was employed as the standard value for computing the solvent-accessible surface area. The PyMOL plugin GUI, written in Python3, was used to calculate the surface area and volume of the active sites in *Fr*ST.

### Phylogenetic tree

Orthologs were searched using the *Fr*ST sequence against proteins in the Protein Data Bank. The obtained orthologous sequences were aligned using ClustalW with the following parameters: a gap opening penalty of 10 and a gap extension penalty of 0.10 for pairwise alignment and 20 for multiple alignment. The delay-divergent cut-off was set at 30. For phylogenetic analysis, the maximum likelihood statistical method was employed using the Jones–Taylor–Thornton (JTT) model.

### Protein clustering

The HHpred tool was utilized [21]. The initial result from the BLAST search against *Fr*ST was used as the input for the second BLAST search, and the obtained data were subsequently forwarded to CLANS [9] to visualize the sequence similarity clusters. In the CLANS visualization, sequence similarity clusters were represented by connecting lines, where darker and shorter lines indicated a higher sequence similarity between the proteins in those clusters. This allowed for the identification and analysis of remote homologous relationships between *Fr*ST and other proteins.

### Enzyme kinetic assays

The sulfurtransferase activity of *Fr*ST was determined using the method previously described by Sörbo et al. [4]. Thiocyanate formation was monitored as a measure of sulfur transfer, with sulfur containing substrates serving as the sulfur donor, and cyanide as the sulfur acceptor. The reaction cocktail consisted of 100 mM Tris-HCl buffer at pH 8.0, varying concentrations of substrates (0–50 mM), 50 mM potassium cyanide, and 5 μg of the enzyme in a total volume of 500 μL. The reaction was initiated by adding the enzyme and incubated for 30 min at 25°C. To stop the reaction, formaldehyde (15% v/v, 250 μL) was added, followed by the addition of 750 μL of ferric nitrate solution (10 g of Fe(NO_3_)_3_·9H_2_O and 20 mL of 65% v/v HNO_3_ per 150 mL). The thiocyanate concentration was estimated based on the absorbance of the ferric ion–thiocyanate complex at 460 nm using a molar extinction coefficient of 4,700 M^-1^ cm^-1^ [22]. Blank reactions without the enzyme were performed under the same conditions and subtracted from the sample readings. The steady-state kinetics of the enzyme were determined using the Michaelis–Menten equation (GraphPad Prism 9 Software, San Diego, CA, USA). Freshly purified protein was used for all enzyme assays to ensure the optimal activity and accuracy of the kinetic measurements.

The optimal temperature and pH for enzyme activity were investigated in the assay containing 5 μg of purified *Fr*ST and 20 mM of sodium thiosulfate. The optimal temperature was determined to be 4, 15, 25, 37, 42, 50, and 60°C using 100 mM Tris-HCl buffer (pH 8.0). The optimal pH was studied by measuring enzyme activity from pH 5.0–9.0 at 25°C. The buffers used were 50 mM citrate phosphate (pH 5.0–7.0) and 100 mM Tris-HCl (pH 7.0–9.0). All values were determined in triplicate and presented as mean ± SD.

### Liquid chromatography–mass spectrometry (LC-MS)

The acids in the samples were analyzed using a UHPLC (Thermo Fisher Scientific, Vanquish) combined with a quadrupole-orbitrap mass spectrometer (Thermo Fisher Scientific, Q Exactive Focus). The separation was performed on a Hypersil GOLDTM aQ column with a mobile phase composed of 0.1% (v/v) formic acid (dissolved in LC-MS grade water) and neat acetonitrile (LC-MS grade). The gradient elution was conducted at a flow rate of 0.3 mL/min and the eluent composition was programmed as follows: 5% acetonitrile for 0–2 min, 5–100% acetonitrile for 2–14 min, 100% acetonitrile for 14–17 min, and acetonitrile 100–5% for 17–21 min. Mass detection was carried out in negative electrospray ionization (ESI) mode, and the operating parameters were set as follows: a detection range of 65 to 650 m/z, with a resolution of 70,000, spray voltage of −2.50 kV (negative mode), capillary temperature of 320°C, sheath gas flow rate of 40 a.u., auxiliary gas flow rate of 10 a.u., auxiliary gas heater temperature of 300°C, S lens RF level of 50.0, maximum injection time of 200 ms, and an automatic gain control (AGC) target of 1×106.

### Protein Data Bank accession numbers

The coordinates and structure factors of *Fr*ST were deposited in the Protein Data Bank RCSB under accession codes: 8K55 (*Fr*ST_*P1*_) and 8K57 (*Fr*ST_*I21*_)

## Supporting information

S1 Fig Kinetics of *Fr*ST with various sulfur donors. The purified *Fr*ST was incubated with 50 mM potassium cyanide and various concentrations of other substrates. After 30-min incubation, thiocyanate formation was determined. The assays were measured in triplicate. The curves were generated by fitting to the Michaelis-Menten equation. The IUPAC nomenclature of the substrate was shown with parentheses.
(PDF)

S2 Fig Identification of product by liquid chromatography-mass spectrometry (LC-MS). LC-MS obtained from the reaction sample of *Fr*ST incubated with 2-methyl-3-sulfanylpropaonic acid (A) and 3-mercaptoisobutyric acid (B) are shown. Total ion chromatograms (TICs) mass spectrum corresponding to each peak is inserted in the LC chromatograms. [M]^+^, molecular ion.
(PDF)

S3 Fig Comparison of the α8–η2 loop region and the active site between subunit A of *Fr*ST_*P1*_(red) and *Fr*ST_*I21*_ (green). 2F_obs_ - 2F_calc_ electron density map (magenta or orange) of (contoured at 1.0 σ) of the 8α-η2 loop region is shown. The residues around the active site and the α8–η2 loop from each subunit with the same orientation are depicted with sticks.
(PDF)

S4 Fig The flexibility of the 8α–η2 loop of TSTs. The monomers of each protein were superimposed. Residues composing the 1–α3 loop and 8α–η2 loop of each protein are highlighted with different color codes.
(PDF)

S1 Table X-ray diffraction data collection and refinement statistics. (PDF)

S2 Table P-values for pairwise T-test comparisons between results from different combination of data using GraphPad Prism 9 Software. (PDF)

## Abbreviations

PAMC: Polar and Alpine Microbial Collection
TST: thiosulfate sulfurtransferase
MST: 3-mercaptopyruvate sulfurtransferase
PDB: protein data bank
AUC: analytical ultracentrifugation
RMSD: root mean square deviation
