# Supplementary material for: Structural and functional characterization of sulfurtransferase from Frondihabitans sp. PAMC28461
Source: PLoS One. 2024 Mar 25;19(3):e0298999. doi: 10.1371/journal.pone.0298999 (PMC10962793; doi:10.1371/journal.pone.0298999)
Supplement: S1 Fig — The purified FrST was incubated with 50 mM potassium cyanide and various concentrations of other substrates. After 30-min incubation, thiocyanate formation was determined. The assays were measured in triplicate. The curves were generated by fitting to the Michaelis-Menten equation. The IUPAC nomenclature of the substrate was shown with parentheses. (PDF) [file pone.0298999.s001.pdf]

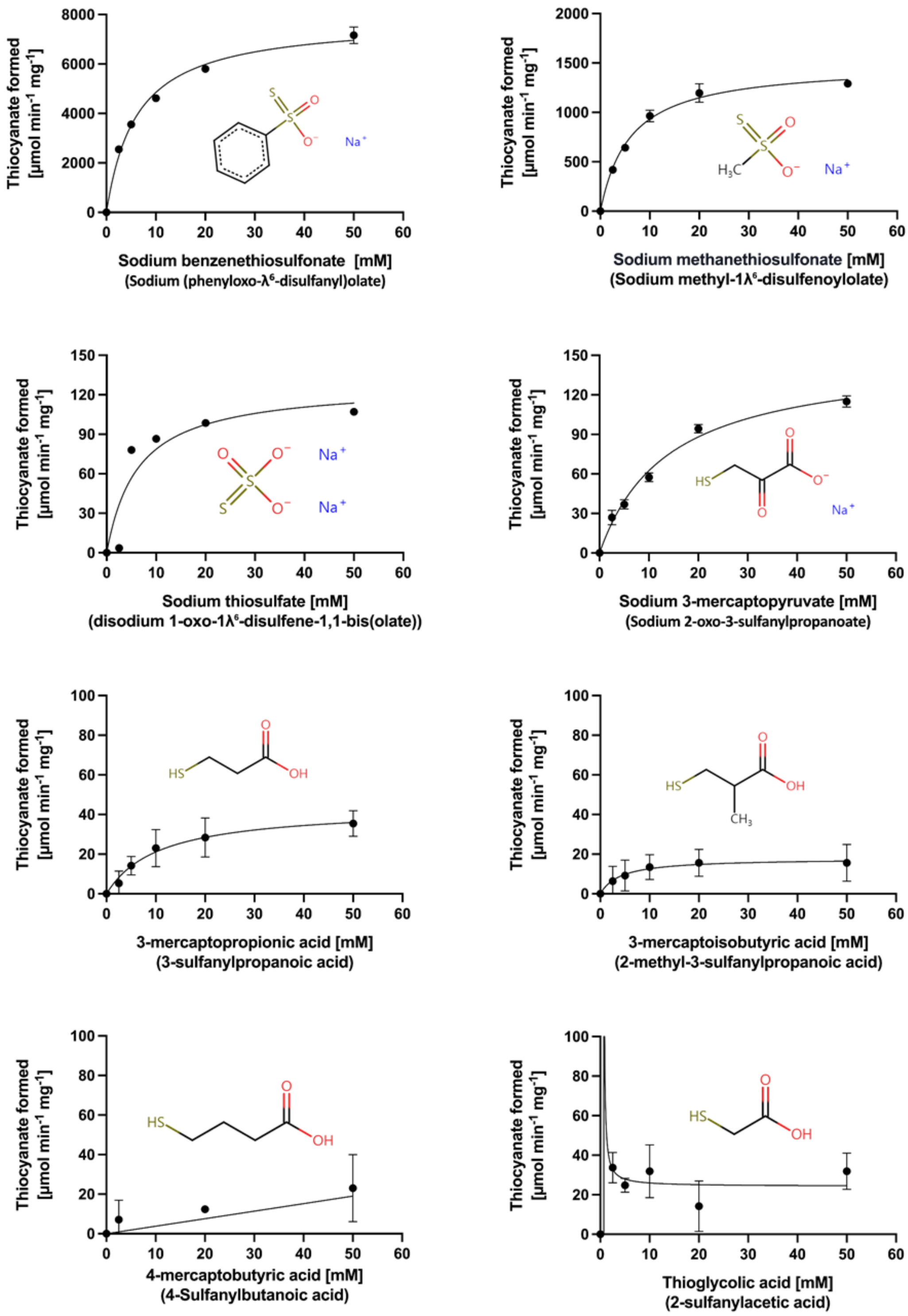

S1 Fig. Kinetics of *Fr*ST with various sulfur donors. The purified *Fr*ST was incubated with 50 mM potassium cyanide and various concentration of other substrates. After 30-min incubation, thiocyanate formation was determined. The assays were measured in triplicate. The curves were generated by fitting to the Michaelis-Menten equation. The IUPAC nomenclature of the substrate was shown with parentheses.
