# Supplementary material for: Structural and functional characterization of sulfurtransferase from Frondihabitans sp. PAMC28461
Source: PLoS One. 2024 Mar 25;19(3):e0298999. doi: 10.1371/journal.pone.0298999 (PMC10962793; doi:10.1371/journal.pone.0298999)
Supplement: S2 Fig — LC-MS obtained from the reaction sample of FrST incubated with 2-methyl-3-sulfanylpropaonic acid (A) and 3-mercaptoisobutyric acid (B) are shown. Total ion chromatograms (TICs) mass spectrum corresponding to each peak is inserted in the LC chromatograms. [M]+, molecular ion. (PDF) [file pone.0298999.s002.pdf]

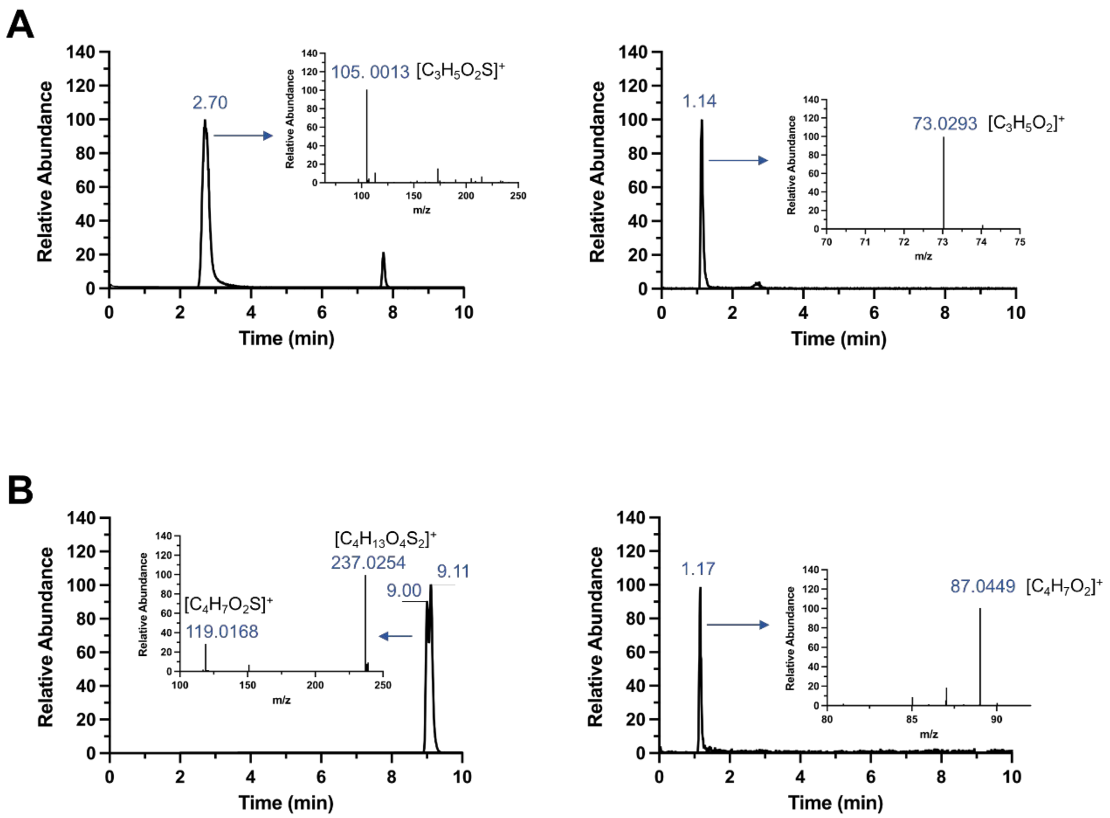

S2 Fig. Identification of product by liquid chromatography-mass spectrometry (LC-MS). LC-MS obtained from the reaction sample of *Fr*ST incubated with (A) and 3-mercaptoisobutyric acid (B) are shown. Total ion chromatograms (TICs) mass spectrum corresponding to each peak is inserted in the LC chromatograms. $[M]^+$, molecular ion.
