# Supplementary material for: Structural and functional characterization of sulfurtransferase from Frondihabitans sp. PAMC28461
Source: PLoS One. 2024 Mar 25;19(3):e0298999. doi: 10.1371/journal.pone.0298999 (PMC10962793; doi:10.1371/journal.pone.0298999)
Supplement: S3 Fig — 2Fobs - 2Fcalc electron density map (magenta or orange) of (contoured at 1.0 σ) of the 8α-η2 loop region is shown. The residues around the active site and the α8–η2 loop from each subunit with the same orientation are depicted with sticks. (PDF) [file pone.0298999.s003.pdf]

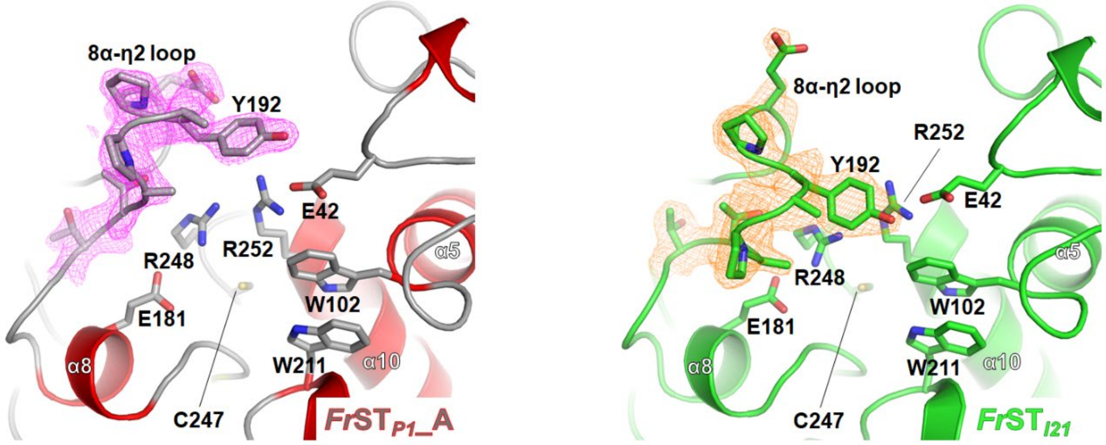

S3 Fig. Comparison of the α8–η2 loop region and the active site between subunit A of *Fr*ST$_{P1}$(red) and *Fr*ST$_{I21}$ (green). 2F$_{obs}$ - 2F$_{calc}$ electron density map (magenta or orange) of (contoured at 1.0 σ) of the 8α-η2 loop region is shown. The residues around the active site and the α8–η2 loop from each subunit with the same orientation are depicted with sticks.
