# Supplementary material for: Structural and functional characterization of sulfurtransferase from Frondihabitans sp. PAMC28461
Source: PLoS One. 2024 Mar 25;19(3):e0298999. doi: 10.1371/journal.pone.0298999 (PMC10962793; doi:10.1371/journal.pone.0298999)
Supplement: S4 Fig — The monomers of each protein were superimposed. Residues composing the 1–α3 loop and 8α–η2 loop of each protein are highlighted with different color codes. (PDF) [file pone.0298999.s004.pdf]

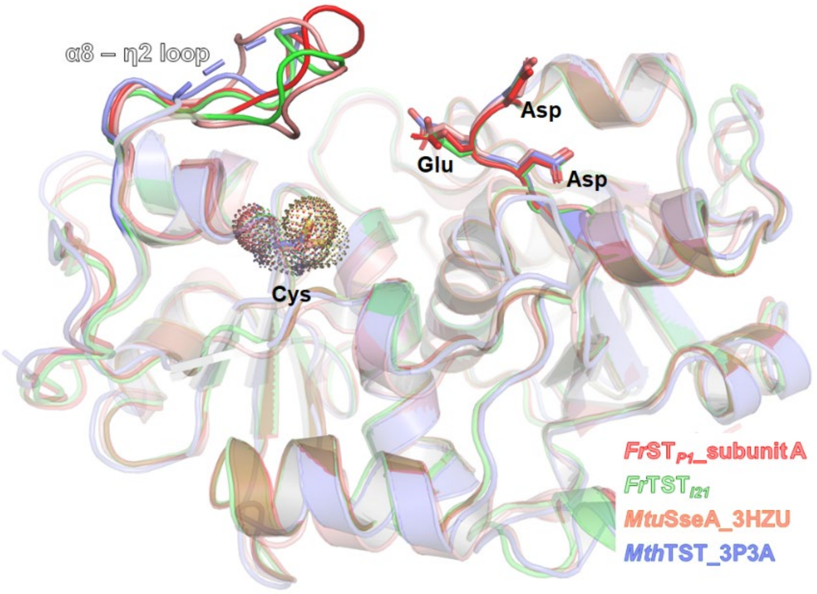

S4 Fig. The flexibility of the 8α–η2 loop of TSTs. Monomers of each protein were superimposed. Residues composing the 1–α3 loop and 8α–η2 loop of each protein are highlighted with different color codes.
