# Supplementary material for: Structural and functional characterization of sulfurtransferase from Frondihabitans sp. PAMC28461
Source: PLoS One. 2024 Mar 25;19(3):e0298999. doi: 10.1371/journal.pone.0298999 (PMC10962793; doi:10.1371/journal.pone.0298999)
Supplement: S1 Table — (PDF) [file pone.0298999.s005.pdf]

S1 Table. X-ray diffraction data collection and refinement statistics.

**Data set**	*Fr*ST$_{P1}$	*Fr*ST$_{I21}$
X-ray source	BL-5C beamline	BL-5C beamline
Space group	*P*1	*I*2$_1$
Unit-cell parameters (Å, °)	a=35.25, b=61.60, c=74.08, α=89.06, β= 76.30, γ=79.64	a=87.47, b=34.76, c=93.37, α= γ=90.00, β=105.72
Wavelength (Å)	0.9794	0.9794
Resolution (Å)	28.91–2.00 (2.05–2.00)	29.13–2.38 (2.47–2.38)
Total reflections	139,022 (10,645)	40,732(4,039)
Unique reflections	38,820 (2,870)	11,072 (1,140)
Average I/σ (I)	10.0 (3.8)	8.4 (2.9)
R_{merge}[a]	0.07 (0.28)	0.09 (0.35)
Redundancy	3.6 (3.7)	3.7 (3.5)
Completeness (%)	96.5 (95.7)	99.3 (98.0)
**Refinement**		
Resolution range (Å)	38.19–1.91 (1.96–1.91)	29.15–2.38
No. of working set reflections	36,884 (2,712)	10,440 (735)
No. of test set reflections	1,935 (154)	631 (46)
No. of atoms	4,679	2,361
No. of water molecules	339	36
R_{cryst}[b]	0.23 (0.29)	0.18 (0.27)
R_{free}[c]	0.26 (0.32)	0.26 (0.42)
r.m.s. bond length (Å)	0.013	0.013
r.m.s. bond angle (°)	1.639	1.639
Average B value (Å^2) (protein)	26.99	37.86
Average B value (Å^2) (solvent)	28.32	29.09
**Ramachandran plot**		
Favored (%)	93.45	94.28
Allowed (%)	4.03	3.70
Outliers (%)	2.52	2.02

[a] $R_{merge} = \sum | \langle I \rangle - I | / \sum \langle I \rangle$.

[b] $R_{cryst} = \sum | |Fo| - |Fc| | / \sum |Fo|$.

[c] R_{free} calculated with 5% of all reflections excluded from refinement stages using high-resolution data. Values in parentheses refer to the highest resolution shells.
