# Supplementary material for: Structural and functional characterization of sulfurtransferase from Frondihabitans sp. PAMC28461
Source: PLoS One. 2024 Mar 25;19(3):e0298999. doi: 10.1371/journal.pone.0298999 (PMC10962793; doi:10.1371/journal.pone.0298999)
Supplement: S2 Table — (PDF) [file pone.0298999.s006.pdf]

S2 Table. P-values for pairwise T-test comparisons between results from different combination of data using GraphPad Prism 9 Software.

**Test set**	p-value [a]	p-value summary [b]	t, df
4°C vs 15°C	<0.0001	****	t=4615, df=2
4°C vs 25°C	0.1993	ns	t=1.890, df=2
4°C vs 37°C	0.2256	ns	t=1.731, df=2
4°C vs 42°C	0.1295	ns	t=2.501, df=2
4°C vs 50°C	0.3204	ns	t=1.310, df=2
4°C vs 60°C	0.0377	*	t=5.000, df=2
15°C vs 25°C	0.039	*	t=4.915, df=2
15°C vs 37°C	0.013	*	t=8.674, df=2
15°C vs 42°C	0.0229	*	t=6.497, df=2
15°C vs 50°C	0.12	ns	t=2.620, df=2
15°C vs 60°C	0.0051	**	t=13.98, df=2
25°C vs 37°C	0.6346	ns	t=0.5552, df=2
25°C vs 42°C	>0.9999	ns	t=0.000, df=2
25°C vs 50°C	0.8925	ns	t=0.1529, df=2
25°C vs 60°C	0.082	ns	t=3.274, df=2
37°C vs 42°C	0.6346	ns	t=0.5552, df=2
37°C vs 50°C	0.4221	ns	t=1.001, df=2
37°C vs 60°C	0.0391	*	t=4.910, df=2
42°C vs 50°C	0.8925	ns	t=0.1529, df=2
42°C vs 60°C	0.0494	*	t=4.330, df=2
50°C vs 60°C	0.0668	ns	t=3.672, df=2

[a] Two-tailed test

[b] Asterisks represent level of significance based on two sample t-test assuming unequal variance (ns, P> 0.05; *, P ≤ 0.05; **, P ≤ 0.01; ***, P ≤ 0.001; ****, P < 0.0001).
